# Radiotracer Technology in Mixing Processes for Industrial Applications

**DOI:** 10.1155/2014/768604

**Published:** 2014-01-30

**Authors:** N. Othman, S. K. Kamarudin

**Affiliations:** ^1^Malaysian Nuclear Agency, Bangi, Kajang, 43000 Selangor, Malaysia; ^2^Department of Chemical and Process Engineering, Universiti Kebangsaan Malaysia (UKM), Bangi, 43600 Selangor, Malaysia

## Abstract

Many problems associated with the mixing process remain unsolved and result in poor mixing performance. The residence time distribution (RTD) and the mixing time are the most important parameters that determine the homogenisation that is achieved in the mixing vessel and are discussed in detail in this paper. In addition, this paper reviews the current problems associated with conventional tracers, mathematical models, and computational fluid dynamics simulations involved in radiotracer experiments and hybrid of radiotracer.

## 1. Introduction

**Figure 1:**
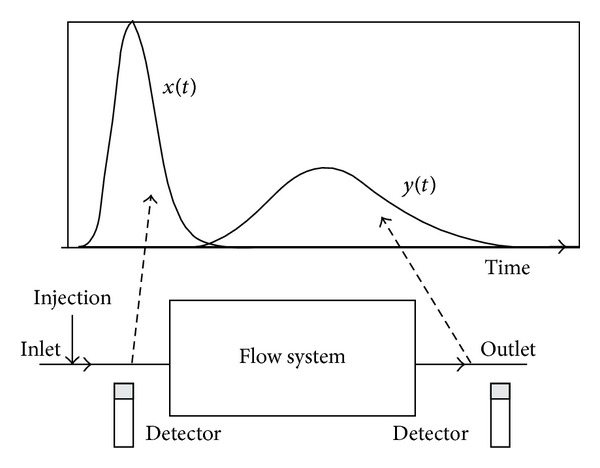
The principle of radiotracer experiment by Furman et al. 2003 [4].

Radiotracers are widely used for the measurement of the flow rate of liquids, gases, and solids in many industrial systems. Thus, in the enhancement of production efficiency and process optimisation, radioisotope-based technology continues to play a rapidly growing role in various industries, such as petrochemicals, oil, and gas, as well as wastewater treatment plants [1–3]. An investigation of many major industrial applications, including fluidised beds, sugar crystallisers, trickle bed reactors, cement rotary kilns, wastewater treatment units, and interwell communications in oil fields, can be performed by injecting a radiotracer at the inlet of the system and monitoring it at the outlet. The data output can be treated and analysed to investigate the behaviour of the system.  Figure 1 shows the fundamentals of a tracer experimental setup as described by Furman et al. [4] in which at least one detector is needed at the inlet to detect the presence of the radioactive source prior to the process investigation and at least one detector is needed at the outlet to detect the radioactive source during the study. The peaks signify the detection of the emitted gamma ray from the radiotracer. Normally, the detector used is a scintillation sodium iodide (Tl) detector.

Radiotracer technology also assists industries in satisfying the critical need for production efficiency through the identification of process malfunctions and anomalies, as well as mechanical damage in the plant. Although radioisotopes have been used to solve a number of industry problems for over 50 years, research and development of the technology continue unchallenged. The greatest benefit of radiotracer technology over the conventional methods is that the investigation can be carried out on-stream and without disrupting the operating process of the plant. Hence, any expensive downtime is avoided and the convenience of direct measurement results in substantial economic benefits and investigating costs. Nevertheless, although the technology is applicable across a broad industrial spectrum, Pant et al. [5] and Pant et al. [6], Hills [1], and Furman et al. [7] stated that the relevant target areas for radiotracer applications are defined and that the most appropriate target beneficiaries of radiotracer applications include the mineral processing sectors, petroleum and petrochemical industries, and wastewater treatment plants. These industries are widespread internationally and are of considerable economic and environmental importance. Moreover, according to the IAEA [2, 3], radiotracer techniques have many advantages, such as high detection sensitivity, in-situ detection, availability of a wide range of compatible radiotracers for different phases, rapid response and high reliability, and accuracy of results.

The measurement of homogeneous mixing efficiency is one of the main applications of radiotracers in the industry. Mixing involves the blending of two or more miscible fluids to obtain a predetermined degree of homogeneity. Stirred tanks are widely used in the process industries to perform many different operations, including the blending of miscible liquids into a single liquid phase, the suspension of solids, the promotion of heat and mass transfer, gas-liquid and liquid-liquid mass transfer, crystallisation, and chemical reactions [8, 9]. Several objectives must be fulfilled when a mechanically stirred vessel is used. Some of these objectives include the homogenisation of single or multiple phases at a specific temperature and concentration of components, which can be affected by the physical properties of the fluids that are being mixed. According to Shekhar and Jayanti [10], the main requirement of the mixing process is to combine two or more fluids that are initially separated. Rahimi and Parvareh [8] observed that, in the liquid phase, the use of impellers and jets are two established methods for fluid homogenisation. Moreover, in the chemical, mineral, and wastewater treatment industries, mechanically stirred tanks are widely used for either simple liquid mixing or for more complex multiphase processes, such as gas-liquid or gas-liquid-solid mixing. Aubin et al. [11] concluded that, to understand the complex phenomena that occur in these mixing tanks, it is necessary to investigate the single- and two-phase flow fields in the vessel and the turbulence characteristics in turbulent applications. Thus, Montante and Magelli [12] suggested an investigation of the flow field that is established in a stirred vessel because it is the most important characteristic of the stirred tank reactor in the processes in which the flow field can affect the homogenisation level. Static mixer, another mixing related service of motionless pipeline devices, is widely used throughout the chemical and hydrocarbon processing industries. This type of mixer is very powerful in the pipeline mixing and is a very dominant option for the laminar flow regime. High reliability over a broad range of flow conditions is achieved when a properly designed static mixer is in operation. Nevertheless, most industrial mixing processes take place in tanks or vessels. Thus, this paper is only concerned with mixing operation in the mixers or vessel.

The residence time distribution (RTD) is one of the important parameters that can provide information on the characteristics of the reactor, such as the flow pattern that occurs [13]. The RTD, which was first developed by Danckwerts [14] has been utilised by many researchers to diagnose possible system malfunctions, such as bypassing, leakage, blockage, channel fouling, and backmixing, and to help estimate the quality of mixing. The RTD, which depends on the flow hydrodynamics and the reactor geometry, influences the chemical reactor performance by affecting a number of reaction properties, such as the conversion and yield. The RTD can be measured by evaluating the concentration of a tracer compound, which is added as a stimulus at the system inlet. A tracer, which has been discussed by Hills [1], the IAEA [2, 3], and Furman et al. [7], has been implemented in experiments that consist of a common impulse-response method in which the tracer is injected at the inlet of the system and the concentration-time curve is recorded at the outlet.

**Figure 2:**
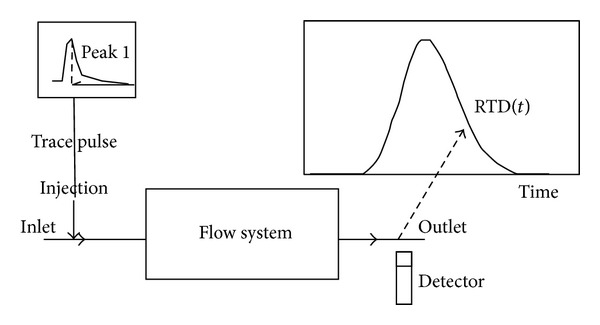
Principle measurement of RTD by Stegowski and Furman (2004) [15].

Stegowski and Furman [15] described the fundamentals of the RTD measurement set up, which is illustrated in Figure 2. The RTD curve of a radiotracer experiment is considered measurable after treatment of the raw data. The treatment of the data involves background correction, radioactive decay correction, starting point correction, filtering, and data extrapolation [16]. Moreover, according to Ding et al. [17] the RTD is a fundamental parameter in reactor design because it can provide information on how long the substrate has been in the reactor and it can help characterise the extent of the deviation in the reactor behaviour from ideal condition. Levenspiel [18] mentioned that the quantified RTD can provide a numerical characterisation of the mixing in a reactor, which helps the process engineer better comprehend the mixing performance of the reactor. In addition, Ding et al. [17] agreed that the dimensionless RTD can potentially be used to compare two different equipment designs and operating conditions.

In addition to the RTD, the mixing time is an important parameter that can be used to determine the homogenisation that occurs in a mixing vessel [16, 19]. There are many definitions of mixing time, which depend on the selected measurement technique. Jafari and Mohammadzadeh [20] defined mixing time as the period of time necessary for a system to achieve the desired level of homogeneity in a given vessel, whereas Bujalski et al. [21] and Patwardhan and Joshi [22] defined mixing time as the time required for the concentration variance to reach a predetermined value. Pramparo et al. [23] and Wang et al. [24] measured the mixing time through the time monitoring evolution of the concentration of a tracer. The tracer can be a chemical species, any substance that can be tracked, or a thermal disturbance, and the measurement techniques that have been used include liquid crystal thermograph, visual observation [21, 25], conductivity, and laser-induced fluorescence [24, 26, 27].

## 2. Current Problems with Conventional Tracers

**Figure 3:**
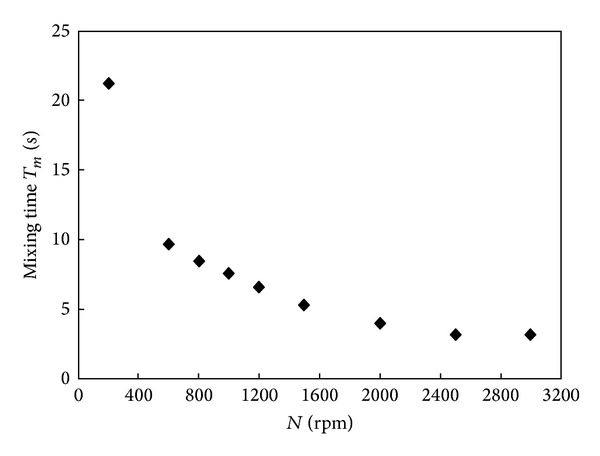
Correlation of the mixing time with the impeller speed by Pramparo et al. 2008 [23].

Extensive studies have been conducted to study the efficiency and flow characteristics of mixing vessels using a nonradiotracer approach. The techniques have been successfully implemented and published, but there are some discrepancies when these are compared with radiotracer techniques. Jafari and Mohammadzadeh [20] and Wang et al. [24] measured the mixing time in a vessel by monitoring the injected tracer concentration as a function of time. These researchers found large deviations in the mixing time between the different measurement locations and detection methods used and concluded that the mixing time was dependent on the location and the detection of the tracer. According to them, this phenomenon was due to the removal of small vortexes, which increases the circulation speed of the liquid vortex. Pramparo et al. [23] described the evolution of mixing time as a function of the impeller rotational velocity, as shown in Figure 3, and indicated that the impeller speed is directly proportional to the mixing time.

**Figure 4:**
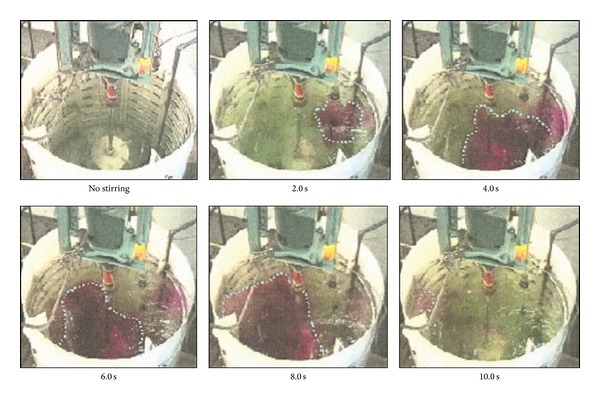
Visualized acid-alkali reactive tracer mixing using the caustic soda-hydrochloric acid-phenolphthalein system conducted by Wabo et al. 2004 [25].

**Figure 5:**
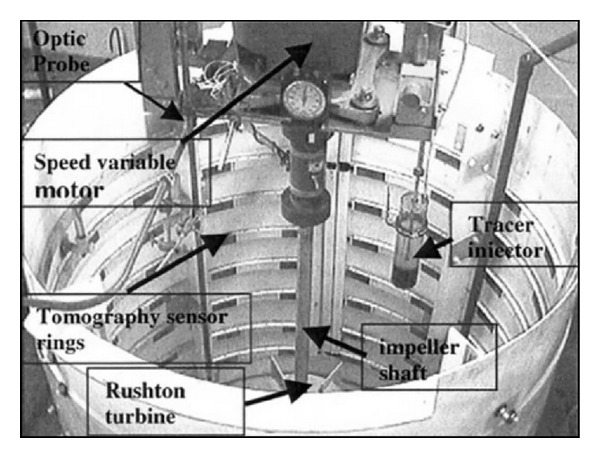
The 8-plane, 16-electrode tomography sensor system fitted for ERT measurement by Wabo et al. (2005) [25].

There is another drawback associated with the use of nonradioactive tracers. Delvigne et al. [27] used a thermal method to calculate the mixing time in which the conductivity probes, or thermocouples, were attached onto the baffles of the vessel. However, due to the size constraint of the probe, which was 0.45 mm in diameter, only a limited area was covered. Therefore, the results failed to represent approximately 90% of the mixing vessel volume [20, 28] and the measured mixing time obtained in this experiment was only 85%. Another technique for the measurement of the mixing time is the use of reagent visualisation. Wabo et al. [25] visualised, in 3D, an acid-alkali reactive tracer in a typical batch stirred vessel reactor, whereas Bujalski et al. [21] calculated the mixing time using the decolourisation of starch. The results of the experiments that were carried out using the thermal method failed to represent approximately 90% of the mixing vessel; only 70% of the fluid volume can be observed through the visualisation by phenolphthalein using a single video camera, as was reported by Wabo et al. [25] and shown in Figure 4. Wabo and his coworkers also used the electrical resistance tomography (ERT) system following the adjacent pair protocol to visualise the fluid mixing during a chemical reaction. They concluded that ERT can suitably image the mixing of reactive tracers and therefore serve as a potential method for quantification of the macrosegregation of reagents in 3D. A total of 16 equally spaced electrodes were used in this study for the current injection; these form a peripheral ring with the 15 voltage pair measurements, as shown in Figure 5. Arratia et al. [13] and Ding et al. [17] concluded that it is inevitable that the introduction of these probes will affect the flow pattern. In addition, the use of these probes involves a complex setup procedure, as described by Wabo et al. [25]. Therefore, the use of a radiotracer is the preferred stimulus response technique because of its noninvasive application and online monitoring systems, which avoid the shutdown of the plant. In addition, Pant and Yelgoankar [19] also declared that radiotracers often have no competing alternative for troubleshooting full-scale industrial reactors. Thus, the integration of radiotracers with computer simulations resolves the previously mentioned problems.

## 3. Mathematical Models: Correlation and Definitions

### 3.1. Correlation and Definitions: RTD

RTD, or  *E*(*t*), is a probability distribution function that describes the amount of time a fluid element spends inside a reactor. It helps in troubleshooting reactors and characterises the macromixing and flow within a reactor [24]. If an impulse of tracer injected at the inlet of a system at time  *t*  equals 0 and its concentration is measured as a function of time at the outlet,  *E*(*t*)  represents the probability that a trace element has a residence time between the time interval  (*t*, *t* + *dt*)  and is defined as follows:

(1) $$\begin{array}{c} E_{\text{out}}\left(t\right)=\frac{C_{\text{out}}\left(t\right)}{\int_{0}^{\infty }C_{\text{out}}\left(t\right)dt}\;\text{such}\;\text{that}\;\int_{0}^{\infty }E_{\text{out}}\left(t\right)dt=1, \end{array}$$

where *C*_out_(*t*) is the detected output signal. The detected signal is normalised by dividing it by the area under the curve, as shown in (1). The mathematical expression for the first moment in discrete form can be written as follows:

(2) $$\begin{array}{c} Mi=\frac{\int_{0}^{\infty }{t\;C}_{i}\left(t\right)dt}{\int_{0}^{\infty }C_{i}\left(t\right)dt}. \end{array}$$

Thus, the experimental mean residence time (MRT) of the system is calculated as the difference between the first moments of the outlet and inlet response curves, where  *i* = 1  and 2:

(3) $$\begin{array}{c} \text{MRT}\;=\;M_{2}-M_{1}, \end{array}$$

where  *M*_1_  is the moment of the curve monitored at the inlet and  *M*_2_  is the moment of the curve monitored at the outlet. Nevertheless, these results should be treated to remove any noises.

Jafari and Mohammadzadeh [20] stated that the RTD can be described by various models, such as a CSTR with exchange volumes, CSTR with a dead zone, and CSTR with a bypass. These models contain many parameters, including the mean residence time, exchange and bypass flow rates, and volume of the dead zone, which can be varied to fit the experimental data. Nevertheless, the models mentioned above do not consider the flow field within the reactor, which results in nonideal behaviour. Levenspiel [18] also noted that there are two commonly cited methods for analysing the RTD curve; these are the dispersion model and the tanks-in-series model. Burrows et al. [29] also addressed more complex methods for the calculation of the volume by short-circuiting the dead zones within the reactor. The dispersion model is based on the ideal plug flow and accounts for the deviations from the ideal flow that is caused by backmixing or random fluctuations. The dispersion number is calculated from the variance of the RTD curve, as shown below:

(4) $$\begin{array}{c} \sigma^{2}=2\frac{D}{uL\;}-2{\left(\frac{D}{uL}\right)}^{2}\left(1-e^{-uL/D}\right), \end{array}$$

where  *D*/*uL*  is the dispersion number (dimensionless),  *L*  is the length of the reactor,  *x*  is a point along the length of the reactor such that 0 < *x* < *L*, and  *t*  is the mean hydraulic residence time. According to Levenspiel [18] a large dispersion number (greater than 0.2) indicates that the reactor behaviour is similar to a single CSTR, whereas the reactor approximates ideal plug flow if the dispersion number is close to zero. Additionally, Burrows et al. [29] discussed the tanks-in-series model, which can also be used to measure the RTD. This model assumes that the flow through the reactor can be characterised by a series of  *N*  equal-sized CSTRs. A tank approaching plug flow would have a large  *N*  value, of approximately 30 or higher, whereas the value of  *N*  for a completely mixed tank will be one.

### 3.2. Correlations and Definitions: Mixing Time

There are several different correlations that can be used for the prediction of the mixing time in an agitated vessel, which achieve a certain degree of mixture uniformity. Pant et al. (2001) [5] and Bujalski et al. (2002) [21] used a correlation developed by Fasano and Penney (1991) [26] to calculate the mixing time:

(5) $$\begin{array}{c} t_{U}=\frac{-\ln\left(1-U\right)}{1.06N{\left(D/T\right)}^{2.17\;}{\left(T/H\right)}^{0.5}}, \end{array}$$

where  *U*  is the degree of uniformity and 0 < *U* < 1. Moreover, Pant et al. [5] and Bujalski et al. [21] used the following relationship for *t*_95_, which was proposed by Ruszkowski [28]:

(6) $$\begin{array}{c} t_{95}=5.91T^{2/3}\;{\left(\frac{\rho V}{P}\right)}^{1/3}{\left(\frac{T}{D}\right)}^{1/3}, \end{array}$$

where *V* is the liquid volume in the tank and *P* is the power input.

**Figure 6:**
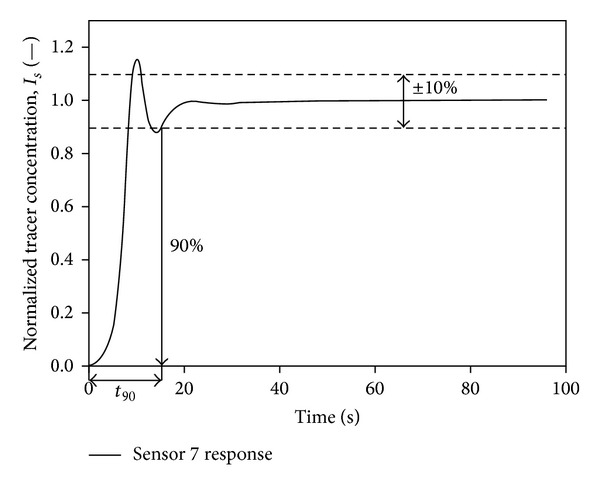
A graphical representation of *t*_90_ mixing time using the simulated concentration response by Bujalski et al. 2002 [21].

**Figure 7:**
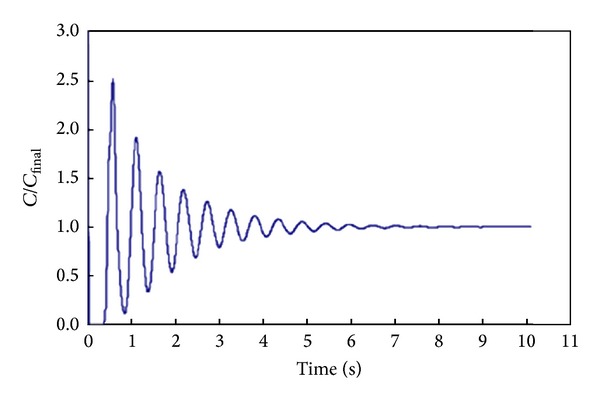
Time evolution of the normalized concentration averaged on the monitor surface by Pramparo et al. 2008 [23].

Bujalski et al. [21] graphically represented the  *t*_90_  mixing time using the normalised simulated concentration response, as shown in Figure 6. This figure showed that the homogeneity of mixing fluid was obtained at 90% degree of uniformity after tracer injection. The normalized tracer concentration indicates that each collected data were divided by summation of collected data as the denominator. Nevertheless, the values greater than one were due to fluctuations of parasitic signals which were not eliminated or treated earlier as suggested by IAEA 2008 and Kasban et al. prior to RTD determination [3, 16]. Moreover, Pramparo et al. [23] stated that the mean mixing time can be obtained by determining the time between two consecutive peaks from the time-variation curve, as shown in Figure 7. Consider

(7) $$\begin{array}{c} UR_{c}=\frac{C_{\infty }-C\left(i,j,t\right)}{C_{\infty }-C_{0}},\\ \theta_{\text{mix}}=\frac{1}{m}\;\sum_{i=1}^{m}\left[\frac{1}{n}\;\sum_{j=1}^{n}t_{95}\left(i,j\right)\right],\\ N\theta_{\text{mix}}=5.98N_{q^{-1/3}}\;{\left(\frac{T}{D}\right)}^{2}. \end{array}$$

The above correlations were derived by Zadghaffari et al. [30] to measure the mixing time required for a particular point to reach 95% of the concentration in the tank. However, the direct comparison of the mixing times of two reactors is only possible if the value of  *Y*  for the two reactors is the same. Thus, Jafari and Mohammadzadeh [20] suggested that the desired degree of homogeneity should be defined at a convenient value of  *Y*  (e.g., 0.90 or 0.95). The mixing in the reactor to achieve a specific level of homogeneity can be expressed in terms of the degree of mixing, or  *Y*:

(8) $$\begin{array}{c} Y=\left|\frac{C_{\left(t\right)}-C_{0}}{C_{\infty }-C_{0}}\right|, \end{array}$$

where *C*_0_ and *C*_*∞*_ are the initial and final average uniform tracer concentrations, respectively, and  *C*(*t*)  is the tracer uniform concentration in the vessel at time  *t*.

## 4. Mathematical Model in Radiotracer Technology

Extensive radiotracer experiments have been carried out successfully in various industries, which indicate the survival and reliability of radiotracers in hostile environments. To verify its feasibility, the IAEA has developed six mathematical models to analyse the experimentally obtained radiotracer curves. The six proposed models are the axial dispersion plug flow model, the axial dispersed plug flow with exchange model, the perfect mixers in series model, the perfect mixers in series with exchange model, the perfect mixers in parallel model, and the perfect mixers with recycle model [3, 16]. The optimised model curve that best fits the experimental curve is chosen.

A series of radiotracer experiments for the measurement of the RTD, mixing time, and flow rate was conducted by Kasban et al. [16] using 300 *μ*Ci^99^Mo as the radioactive tracer, which was added at a height of 800 mm in the mixing vessel. Four paddle impellers were installed and pretreatment of the raw data was carried out using Matlab. The IAEA software was utilised to characterise the RTD mathematical models that were recommended by the IAEA. The perfect mixer with recycle model best represented the curve of the experimental data and the RTD was found to be 57 min. A flow rate of approximately 8.75 Ls^−1^ was used to accommodate the 175 L of water in the flow rig system. The authors concluded that the speed of the impeller affects the mixing time required for the system to reach homogeneity. However, the authors did not describe the calculation of the mixing time or the malfunction that was identified from the findings. They also did not accurately describe the RTD experimental setup and the mixing time measurement. In addition, the authors did not clarify the impeller size and geometry and the parameters used in the mixing process optimisation.

Radiotracers have also successfully assisted industries in the research of multiphase flow. Sugiharto et al. [31] determined the RTD and the system flow rate in a 24 in. multiphase flow hydrocarbon transmission pipeline containing approximately 95% water, 3% crude oil, 2% gas, and negligible solid material. Nevertheless, the types of radiotracer sources used in this experiment were different; I-13 and Na-24 were used independently for the measurement of the RTD in hydrocarbons and water, respectively. In this instance, the tanks-in-series model best described the RTD of the system. The authors also discovered that the water moved faster than the hydrocarbon even though the density of the water is higher. This might be because water is more dominant in the transmission line and because the movement of the crude oil is slowed by friction with gas at the top layer and friction at the water-crude oil interface. Moreover, Behin and Aghajari [32] studied the RTD measurement in a pilot-scale oil-water separator operated by Drood oil of the Iranian Offshore Oil Company (IOOC) located in Kharg Island using 5mCi I-131 as sodium iodide and 4mCi of I-131 as iodobenzene for the aqueous and organic phase, respectively. The separation of the crude oil and water mixtures is an important process in the oil and chemical industries. The researchers reported that the experimental results were in good agreement using the model of perfect mixing tanks-in-series (with a dead zone) to describe the liquid behaviour. The models obtained in these two case studies were both tanks-in-series models. It can therefore be concluded that tanks-in-series models suited the multiphase flow profile.

The next case studies, which were conducted by Pant et al., indicate the superiority of radiotracer technology in hostile industrial environments. In addition, each case study developed a mathematical model that fit the experimental results satisfactorily although the chosen models varied between applications. Pant et al. [33] conducted an RTD study in a pilot and an industrialised fluid catalytic cracking unit (FCCU) using an intrinsic tracer. The identified tracers were lanthanum-140 and sodium-24 and were obtained from the catalyst sample. The tracers were characterised using neutron activation analysis to investigate the degree of axial mixing and radial distribution in the riser section of the FCCUs and to determine the residence time distribution of the catalyst. The axial dispersion model (ADM) was used to represent the actual RTD obtained from the radiotracer experiments. According to Pant et al., the ADM is the best model to describe material flow in a tabular reactor, such as FCCU, because, according to Kasban et al. [16] this model can describe one-dimensional convection and dispersion in a pipe. Moreover, Pant et al. [6] measured the RTD of coal particles in a pilot-scale fluidised bed gasifier (FBG). These researchers successfully used 100 g of each lanthanum-140- and gold-198-labelled coal particles or 100 g of lanthanum-140- and gold-198-labelled coal particles (50 g of each) at a very high temperature (1000°C) as the radiotracer source. They represented the behaviour of the coal particles that flow from the bottom of the gasifier with tanks-in-series model. The results revealed that there was a good degree of mixing and that only a small fraction of the feed material bypassed and short-circuited from the bottom of the gasifier. This was also the first report of the use of radiotracers in an FBG in India and proved the superiority of the radiotracer techniques in the energy industry.

**Figure 8:**
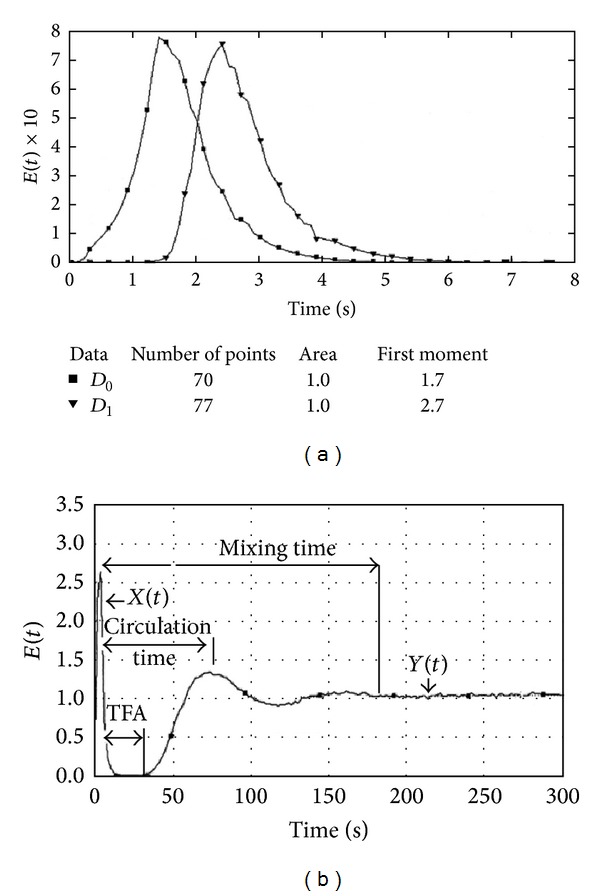
(a) Flow rate measurement; (b) time of first appearance, circulation, and homogenisation or mixing time by Pant et al. 2001 [5].

Pant et al. [5] also determined the RTD in a sludge hygienisation research irradiator (SHRI) using 7–10 mCi NH_4_^82^Br as the tracer to troubleshoot the malfunction in the reactor. Their results disclosed the presence of a dead zone in approximately one-fourth of the irradiator with low flow rates and a semistagnant volume and slow flow exchange with higher flow rates. In this study, the tracer first appearance time (TFAT) was implemented to estimate the minimum dose received by the sludge from one circulation through the irradiator. The authors discussed the methods used to determine the flow rates, circulation time, and homogenisation time (mixing time) from the derived data, as shown in Figure 8. The three models used to measure these three reactor characteristics were the models of ideal mixers in series with plug flow in a recirculation loop, ideal mixers in series with plug flow inside the irradiator and in a recirculation loop, and ideal mixers in series with stagnant volume and plug flow in a recirculation loop, respectively. All of these model simulations provided important information about the hydrodynamic behaviour of the closed recirculation, batch-type sludge irradiator. In addition, the case studies that were conducted by Pant et al. showed the ability of radiotracer technology not only to survive in hostile industrial environments but also to troubleshoot and pinpoint the malfunctions or anomalies in the plants of different industries. The good agreement that was obtained between the developed models and the experimental results indicate the accuracy of the theories.

Moreover, Pant and Yelgoankar [19] investigated the RTD in servotherm special oil as the heat transfer medium (HTM) in two identical aniline production reactors, one of which was the reference reactor. These researchers used ^82^Br as para-dibromo benzene as the radioactive source. The output from the RTD indicated the presence of undesired parallel flow streams in the shell-side of the abnormal reactor due to a 60% fouling of the reactor. The data were treated prior to any RTD analysis to ensure that only the radioactive material was analysed. The authors implemented tanks-in-series model to simulate the results and detected the presence of several anomalies in the reactor, which were mainly fouling/scaling or dead volume. Due to the large amount of fouling in the reactor and the possibility of the occurrence of parallel streams in the shell-side of the reactor, they implemented a model of tanks-in-series with two parallel streams to represent the RTD curve that was obtained from the radiotracer experiments. The results showed the ability of the radiotracer technology to highlight the percentage of abnormalities that were present in the processing plant accurately, which was impossible to achieve with other conventional methods.

The next case studies demonstrate that radiotracer technology can be used in any form, that is, solid, gas, or liquid. The form of the radiotracer source is based upon the medium of the plant under investigation to obtain superior physicochemical compatibility. Santos and Dantas [34] determined the RTD by calculating the transit of the methylbromide gaseous tracer and the nondispersive Co-60 tracer in an FCC cold mode. They found good agreement between the Experimental Cold Model (ECM) and the RTD results obtained from the radiotracer experiments. Moreover, Klusener et al. [35] investigated the RTD in a horizontal cross-flow bubble column reactor of a commercial ethylbenzene oxidation plant using Ar-41 gas as the tracer. These researchers implemented the axial dispersion and the tanks-in-series models to describe the mean residence time, the number of tanks,  *N*, and the Peclet number. The results showed that the ADM described the gas radiotracer experiments better than the tanks-in-series model. This result makes sense because the ADM describes one-dimensional convection and dispersion in a pipe more accurately than the tanks-in-series model. Thus, the RTD results enabled the plant to implement extensive changes to optimise the plant yields and improve the selectivity at a lower reaction temperature.

Meanwhile, Furman et al. [4] conducted a radiotracer experiment on a ball mill in which the grinding of copper ore occurs. In this study, they used the radioactive isotopes ^64^Cu and NH_4_Br (^82^Br) as the tracers for the copper sulphurs and water, respectively. They suggested a series of perfect mixers with dead volume to represent the radiotracer experiments. However, in this case study, it was necessary to use a different model to accurately fit the experimental data from the radiotracer experiment. Therefore, the optimised model consists of a serially connected plug flow reactor and perfect mixers with dead volume.

Stegowski and Furman [15] highlighted different RTD models that are commonly used to represent flow patterns of industrial systems in the Laplace s-domain, as shown in Table 1. They measured the RTD in a copper ore thickening, filtration, and drying system using 1 GBq of Cu-64 as the radioactive tracer. The RTD study shows the presence of a dead zone and bypasses, which affect the efficiency of the dewatering system, and provides a detailed analysis of the solid state behaviour inside the dewatering subsystems. The parameters involved for each model are highlighted in the table. In this case study, the main parameters for the plug flow and perfect mixing series model are the residence time, the mean residence time, and the number of perfect mixers.

The next case study shows the capability to integrate the mathematical RTD model with another model and indicates that the models recommended by the IAEA do not have to be used independently to describe a process but can be integrated easily with other models. In addition, the model can also be used to describe the present anomalies in the reactor or system under study. Klusener et al. [35] studied the influence of the inlet positions on the flow behaviour inside a photoreactor using 1 mCi^113m^In. Initially, the flow behaviour was modelled using the small plug flow and perfect mixing cell model, which represents the jet effect. Nevertheless, to represent the whole system, the optimised model consisted of two parameters: the mean residence time in the plug flow reactor and the mean residence time in the perfect mixing cell. The optimised models described the experimental results satisfactorily. To simulate the lateral inlet configurations, the author used the plug flow with axial dispersion model, which uses the mean residence time and the Peclet number as the parameters. The results showed that there was a large stagnant volume in the central inlet, which was reflected in the estimation of the first moments of the RTD curves. According to Nigam et al. [36] new data on backmixing and mass transfer parameters have been obtained from the residence time distribution (RTD) of a liquid in an air-water concurrent down flow trickle-bed reactor that contains six different types of alumina-support porous catalysts, that is, spheres, tablets, holed tablets, and extrudates. They used 10–20 MBq of sodium pertechnatate salt samples containing radioactive ^99m^Tc to conduct the RTD studies. They implemented a comprehensive axial dispersion exchange and intraparticle diffusion RTD model to interpret the effect of the gas and liquid input flow rates on the liquid axial dispersion coefficients, the liquid-liquid mass transfer coefficients between the stagnant and dynamic liquid zones, and the liquid-solid mass transfer coefficients between the static liquid and the subtended wetted fractional pellet areas.

The typical use of radiotracer approaches is to compare the experimental curves with the RTD mathematical models that are proposed by the IAEA. Nevertheless, there are some papers that do not mention the mathematical model that best describes the radiotracer experiments. Instead, they simply directly highlight the malfunction of the investigated system or vessel. Oriol et al. [37] investigated the feasibility of using the gas radiotracer Krypton-81 m to diagnose the flow maldistribution in a multiphase heat exchanger in which the horizontal pipe was fed by an air-water mixture. Both smooth and perturbed curves were observed throughout the experiments. The curve shape indicates whether the two-phase flow regime is continuous, which can be stratified, annular or wavy, or dispersed, which is represented by plug, slug, or bubbles. The studies indicated that the shape of the signal can be used to determine the gas-liquid regime. Very high frequencies are associated with a bubble flow regime, whereas smaller frequencies are associated with slug or plug flow regimes. Moreover, Abellon et al. [38] studied the residence time or solid circulation rate of a single particle in an interconnected fluidised bed facility (IFB) with glass beads using a glass particle labelled with radioactive ^24^Na or ^92^Ir. The ^92^Ir was produced by melted glass and the addition of 0.05 wt% iridium. In this study, the radiotracer particle size was varied to determine the correlation between the radiotracer particle size and the MRT while all other parameters were held constant. The researchers found that the MRT of the radiotracer is independent of its size as long as it is within the size range of the batch. They also concluded that all of the particles in the IFB acted as one “homogenous fluid” and fluidised from one cell to another. The previously described models for the different case studies have not provided insights into the processes that occur in the vessels or systems. The industries are mostly interested in understanding the reasons that underline the flow pattern that arises in the plant instead of simply knowing the mathematical model that would represent it or a graphical profile of the flow. Thus, the use of computational fluid dynamics (CFD) would complement the results obtained from the radiotracer experiments.

## 5. Computational Simulations Using CFD

Computational fluid dynamics (CFD) is a computer modelling tool that enables the visualisation of complex processes. According to the IAEA [2] and Ranade [39], CFD is a superior and predictive analysis that provides clear spatial pictures of the process under study, which includes information such as flow patterns and velocity maps. Moreover, a number of industrial reactor engineering applications utilise commercial CFD tools to ensure enhanced maintainability. The ability to visualise and monitor the flow pattern in a chemical process plant results in a better understanding of the actual chemical process. Therefore, this increased ability to monitor flow patterns will promote the use of radiotracer technology in many industries, especially a number of Malaysian industries.

### 5.1. RTD in CFD

Numerous experimental and CFD studies on the RTD in tanks have been carried out and published over the years. Zadghaffari et al. [30] concluded that the experimental validation of the numerical RTD is more convenient than the experimental validation of the complete flow that is obtained numerically. Wang et al. [24], Bai et al. [40], and Furman and Stegowski [41] agreed that the present numerical method used to calculate the mixing time and the residence time distribution in a stirred tank has proven to be reliable and widely applicable. Moreover, the liquid flow and tracer transport can be described by a general partial differential equation in the cylindrical coordinate system in terms of the velocity components  *u*,  *v*,  *w*, the turbulent kinetic energy  *k*, the viscous dissipation *ε*, and the tracer concentration  *c*. Consider

(9) ∂(ρφ)∂t+1r∂∂r(ρurφ)+1r∂y∂θ(ρvφ)+∂(ρwφ)∂z  =1∂r∂r(rΓϕ,eff∂ϕ∂r)+1r∂∂θ(Γϕ,effr∂ϕ∂θ)   +∂∂z(Γϕ,eff∂ϕ∂z)+S,

where *φ* is the dependent variable and *S* is the source term per volume. The eddy diffusivity was modelled using a standard  *k*-*ε*  model and the impeller rotation was modelled using the improved inner-outer iterative procedure [17] with multiple reference frames [24, 29] and a sliding mesh approach [42]. Bai et al. [40] used the realisable  *k*-*ε*  model and multiple reference frames (MRFs) to simulate the stirred tank at steady-state. Furman and Stegowski [41] obtained the reactor RTD from stochastic particle tracking or discrete phase models and used a large number of tracers (*∼*10^5^) to reduce the statistical uncertainty. They concluded that both the standard and the RNG *k*-*ε* models predict the experimental RTD successfully, but a significant deviation was obtained between the simulated and experimentally measured mean residence time and tank space time, which indicates that the stochastic tracking model failed to accurately reproduce the flow behaviour [40].

**Figure 9:**
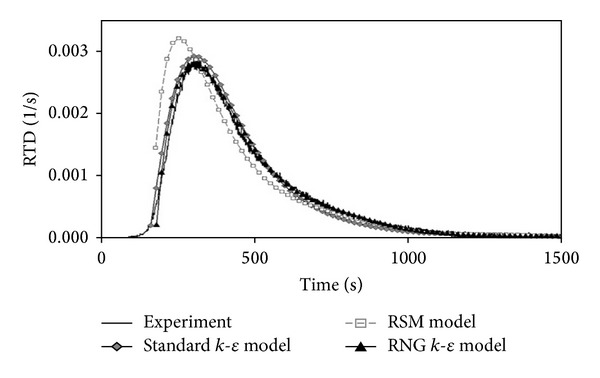
Experimental and simulated RTD functions by Furman and Stegowski 2011 [41].

There are several factors that influence the mean residence time. In a CFD model, the rotational speed does not directly affect the mean residence time. Nevertheless, experimental evidence shows that the mean residence time decreases with increasing rotational speed. The centrifugal force that is generated by a high rotational speed results in the appearance of dead zones near the wall surfaces of the vessel and most of the fluid flow moves directly along the stirring shaft to the outlet. However, as the inlet flow rate increases, a change in the rotational speed does not affect the mean residence time. Cao et al. [43] explained that, to quantitatively understand the RTD, the experimental data have to be fitted to an adequate model, as described in Section 3, to describe the nonideal reactor flow pattern, which will provide a better understanding of the quality of mixing, especially for reactions that are not first order. Moreover, the placement of a stirrer at the bottom of an annular reactor with an optimal speed of 150–250 rpm could narrow the RTD curve and improve the mass transfer for surface reactions. The reactor increasingly behaved as a single stirred tank reactor, which is reflected by the broadening of the RTD curve at higher stirring rates. Furman and Stegowski [41] described an example of curve fitting the RTD data with other models, as shown in Figure 9.

**Figure 10:**
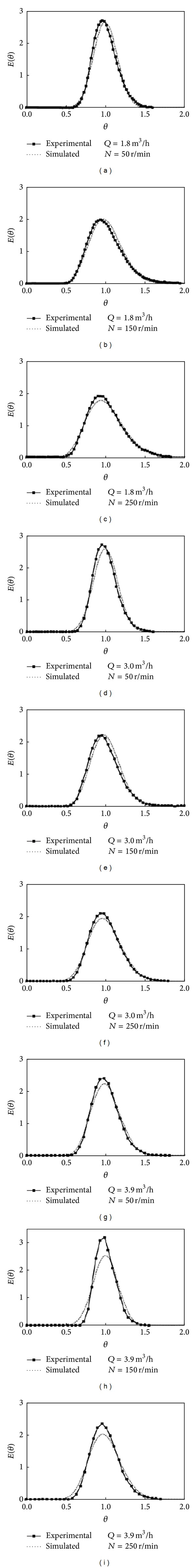
Example of reactor malfunction from RTD curves by Cao et al. 2009 [43].

The detailed description of a reactor malfunction using RTD data is best described by Cao et al. [43] as shown in Figure 10. The  *E*(*θ*)  curves show the presence of stagnant zones. The appearance of the maximum  *E*(*θ*)  at  *θ* < 1 indicates the presence of short-circuits, whereas the appearance of the maximum  *E*(*θ*)  at  *θ* > 1 indicates the presence of backmixing. However, if the maximum of the *E*(*θ*) curve appears near *θ* = 1 at an impeller speed of 50 r min^−1^, the flow pattern approximates ideal plug flow, as illustrated in Figure 10(g). However, if the maximum of  *E*(*θ*)  is offset to  *θ* < 1, the flow is influenced by the presence of short-circuits at an impeller speed of 400 r min^−1^. Sahle-Demessie et al. [44] and Gavrilescu and Tudose [45] concluded that the width of the RTD curve is an appropriate measurement to determine the approximation of the flow pattern to plug flow.

**Figure 11:**
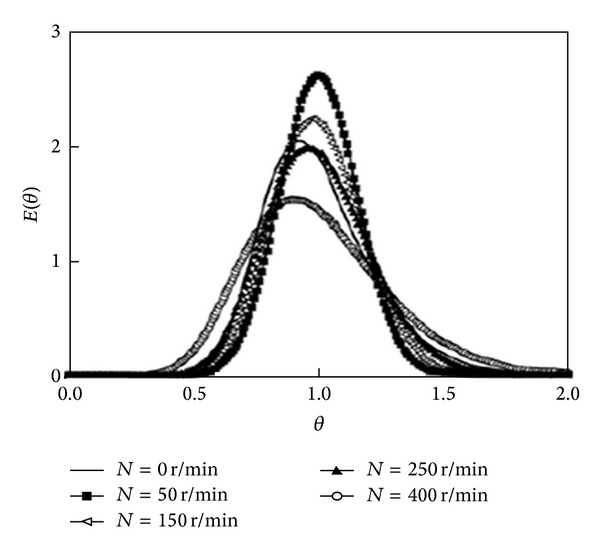
Effects of impeller speed with RTD curves by Cao et al. 2009 [43].

**Figure 12:**
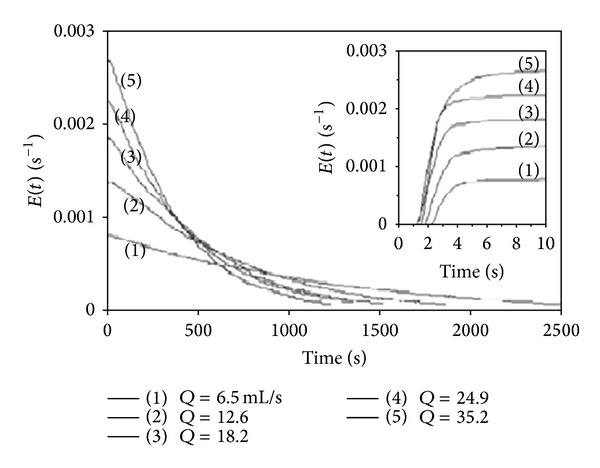
Effects of flow rate with RTD [20].

Another factor that affects the RTD is stirring. Cao et al. [43] explained that the RTD curve is narrow with stirring, which causes the flow to approach plug flow conditions. However, although slow stirring can narrow the RTD curve and improve the performance of the reactor, increasing the impeller speed widens the RTD curve.  Figure 11 shows the numerical RTD curves at different impeller speeds. From the figure, it can be concluded that the RTD curve tends to flatten as the speed of the impeller increases, which indicates that a high impeller speed can also worsen reactor performance. Thus, researchers should conduct preliminary experiments to identify a set of reliable impeller speeds prior to process optimisation. Jaffari and Mohammazadeh [20] studied the effect of the liquid flow rate on the RTD in a gas-induced contactor with a constant impeller speed and liquid volume of 1100 rpm and 10 litres, respectively. Because of the constant impeller speed, it was expected that the values of the maximum concentration [*C*(*t*)] and the corresponding times would be equal. The RTD curve became wider with decreasing liquid flow rates, which increased the mean residence time, as shown in Figure 12. Hence, by increasing the impeller speed, the time corresponding to the maximum point of the RTD curves decreases. It can therefore be concluded that the impeller speed and the liquid flow rate can have a significant effect on the RTD curve.

### 5.2. Mixing Time in CFD

Numerical simulation is another technique that is used to calculate mixing time. CFD is currently widely used to verify experimental results. Osman and Varley [46] measured mixing time in an unbaffled vessel with a Rushton turbine using the moving reference frame (MRF) approach and Jaworski et al. [42] studied homogenisation in a baffled vessel stirred by a dual Rushton impeller using a similar approach. They both predicted a mixing time that was 2-3 times higher than the measured value and attributed this deviation to a wrongly predicted tangential velocity field and an under-prediction of the mass exchange between the recirculation zones that are generated by the turbines. Thus, Bujalski et al. [21] used the transient scalar transport equation in a stationary reference frame for a baffled reactor; however, the mixing time was still over-predicted by a factor of approximately two, which is similar to the results obtained by Jaworski et al. [42]. To directly compare their results with the study performed by Osman and Varley [46] the authors should also attempt to apply the scalar transport equation to an unbaffled vessel. They should also use the low Reynolds in  *k*-*ε*  model, which was successfully used by Shekhar and Jayanti [10] to simulate the flow field and mixing characteristics in an unbaffled vessel stirred with a paddle impeller. The studies conducted by Zadghaffari et al. [47] who used the MRF solution as the starting point and switched to a sliding mesh for the unsteady conditions and Bujalski et al. [21] who implemented a sliding mesh approach concluded that the mixing time depends on the feed point because the inner rotating mesh volume, which was used to model the rotating impeller, was the main factor that affected the distribution of the tracer.

Yianneskis [48] reported that the position of the probe and the tracer injection point do not have a significant effect on the final homogenisation time. Rahimi and Parvareh [8] discovered that the prediction of the mixing using the RNG version of the *k*-*ε* model yielded better results with realisable and standard conditions, whereas Zadghaffari et al. [47] discovered that the use of a sliding mesh technique with the LES turbulent model was faster than the RNG model, but over-predicted the liquid homogenisation time. Nevertheless, Javed et al. [49] used a fully predictive sliding mesh technique and a secondary liquid as a tracer and found that the discrepancy between the predicted and the actual transport of the tracer within a vertical plane was caused by the location where the tracer injection was simulated. Zadghaffari et al. [30] and Zadghaffari et al. [47] agreed that, at given and constant inlet and outlet flow rates, the mixing efficiency decreased at higher agitation speeds because the stronger radial outflow and increasing Reynolds number push the species rapidly into the lower and upper recirculation loops. Hence, the impeller rotational speed increases the liquid pumping capacity of the impeller and thus reduces the mixing time.

## 6. Hybrid of Radiotracer and CFD

The use of CFD modelling in process industries has attracted a lot of attention since 1990. Modelling and numeric simulation have been used to validate a large amount of experimental data, especially in process industries. Rahimi and Parvareh [8] stated that this technique was developed due to the availability of advanced measurement techniques that could be used to validate the theoretical results. Recently, tracer engineers have started to integrate radiotracer experiments with computer simulation to improve the industrial process visualisation and optimisation. The objective of the integration of radiotracer experiments and visualisation modelling is to provide insights into the industrial process and to ensure that the movement of the radiotracer is within safe boundaries. Moreover, the technology provided by the CFD simulations allows the monitoring and tracking of a radiotracer, which will increase the confidence of using radiotracers in plants. Thus, this combination of technologies can provide a significant contribution to the industries because it can be used to localise any anomalies, process malfunctions, or mechanical damages in a plant without requiring its shutdown. Hence, expensive downtime is avoided and the convenience of direct measurements results in lower process investigation costs. Although there is limited literature on the hybrid of radiotracer technology and CFD, a large amount of research is currently being carried out in most IAEA countries on the development of this technology.

Stegowski and Furman [15] used a solution of Na^99m^TcO_4_ as the radioactive source for the investigation of the RTD in a laboratory jet mixer. They compared the experimental RTD derived from the radiotracer experiments with the simulations from three different CFD models: standard *k*-*ε*, RNG *k*-*ε*, and RSM. The authors found that the RNG *k*-*ε*, model best represents the radiotracer experiment curve. However, the authors did not describe the type of RTD mathematical model that they utilised. The authors also failed to highlight the anomalies in the laboratory jet mixer. Another hybrid investigation was conducted by Din et al. [50] who integrated CFD modelling with radiotracer experiments in a two phase counter-current pulsed sieve plate extraction. They used 0.5 mCi Tc-99m and the axial dispersion model to determine the RTD and to study the hydrodynamics of the dispersed phase. They used the RTD data to estimate the proportions of water and kerosene as a continuous phase. They discovered that the axial dispersion model represents approximately 72.17% of the experimental value but it is still acceptable for the study of the hydrodynamics. The CFD models used were multiphase, standard *k*-*ε*, porous media, and pulse generation models. In their paper, the authors described the type of model used, as recommended by the IAEA. However, the results obtained from the CFD simulation were not acceptable because they deviated approximately 30% from the experimental measurements. It is possible that another type of model, such as the RNG or realisable standard *k*-*ε* models, might yield better results.

The combination of radiotracer technology and computer modelling has increased the use of both of these technologies, which indicates its superiority among the conventional techniques that are used worldwide.

## 7. Conclusions

In this review, the development of radiotracer technology has been described. The introduction described radiotracer technology in general, which was followed by a summary of the current problems that arise from the use of conventional tracers. A description of the mathematical models that are used in radiotracer applications was then presented, followed by a summary of computer simulations to analyse the RTD and mixing time and an introduction of a hybrid of radiotracer technology and computational fluid dynamics (CFD). The literature described in this review indicates the versatility and feasibility of radiotracer technology in numerous industrial and laboratory applications. Future research on radiotracer technology will fully utilise and validate its use with numerical solutions in the study of mixing vessels because radiotracer techniques are widely used for the identification of industrial malfunctions and measurement of process parameters, such as the mean residence time (MRT), residence time distribution (RTD), and flow rate. Radiotracer technology has many advantages over conventional tracers; these include its high detection sensitivity, physicochemical compatibility, in-situ detection, and limited memory effect. It is possible that radiotracer technology can be used in combination with an experimental design that implements the Taguchi Orthogonal Array Method, which is a robust design method that was developed to reduce cost and improve the quality of chemical process [51]. The Taguchi method has been applied initially to determine the minimum number of experiments that are required for process optimisation, which is beneficial because of the inclusion of radioactive experiments. This method involves the use of orthogonal array techniques to investigate the simultaneous variation of several parameters and the interactions between parameters.

## Figures and Tables

**Table 1:** Elementary models used in experimental RTD data analysis [4].

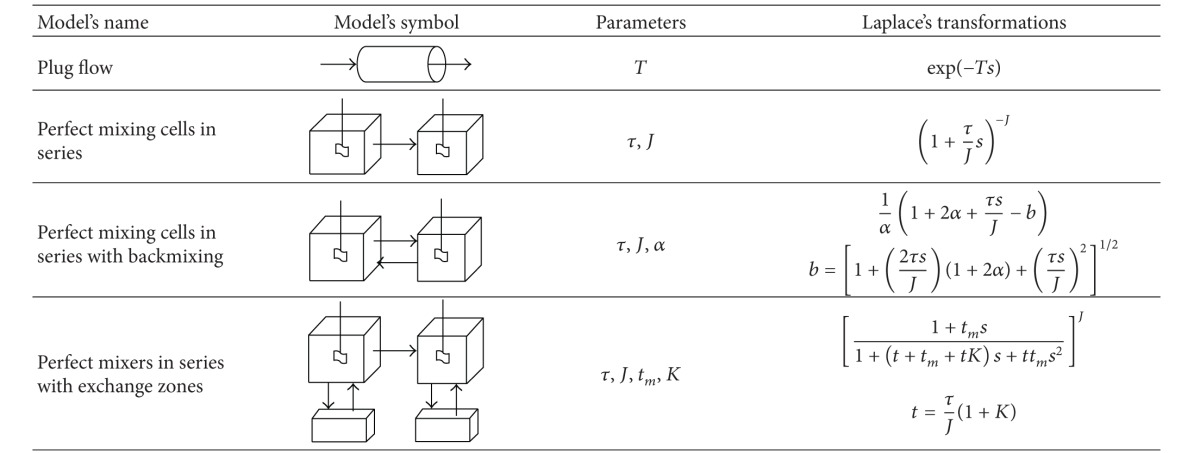

*T*: residence time, τ: mean residence time, *J:* number of perfect mixers, *α*: part of the backmixing flow, *t*_*m*_: mean residence time for exchange zones, *K*: volume ratio between exchange zones and mixing cells.

## Nomenclatures

*C*: Tracer concentration, kg/m^3^
*C*_0_: The tracer initial concentration, kg/m^3^
*C*_*∞*_: Final average uniform concentration, kg/m^3^
*D*: Impeller diameter, m
*D*/*uL*: Dispersion number (dimensionless)
*L*: Blade length, m
*P*: The power input, W
*R*: Radial direction, m
*T*: Tank diameter, m
*T*: Residence time, s
*J*: Number of perfect mixers
*K*: Volume ratio between exchange zones and mixing cells
*U*: The degree of uniformity and 0 < *U* < 1
*k*: Turbulent kinetic energy, m^2^/s^2^
*t*_*m*_: Mean residence time for exchange zone
*u*: Mean velocity vector, m/s
*u*, *v*, *w*: Velocity components, m/s
*V*: The liquid volume in the tank, m^3^
*x*: A general point along the length of the reactor such that 0 < *x* < *L*, m
*z*: Axial coordinate, m.

### Greek Letters

*ε*: Viscous dissipation rate, m^2^/s^3^
*φ*: The dependent variable
*t*: Mean hydraulic residence time, s
*θ*_mix_: Degree of mixing
Γ: Molecular diffusivity, m^2^s^−1^
*ρ*: Liquid density, kg/m^3^
*ϕ*: Tracer fluctuating volumetric fraction
*τ*: Mean residence time, s
*α*: Part of the backmixing flow.
